# Epstein-Barr Virus Infection Alone or Jointly with Human Papillomavirus Associates with Down-Regulation of miR-145 in Oral Squamous-Cell Carcinoma

**DOI:** 10.3390/microorganisms9122496

**Published:** 2021-12-02

**Authors:** Chukkris Heawchaiyaphum, Tipaya Ekalaksananan, Natcha Patarapadungkit, Suchin Worawichawong, Chamsai Pientong

**Affiliations:** 1 Department of Microbiology, Faculty of Medicine, Khon Kaen University, Khon Kaen 40002, Thailand; jukkris.003@gmail.com (C.H.); tipeka@kku.ac.th (T.E.); 2 HPV & EBV and Carcinogenesis Research Group, Khon Kaen University, Khon Kaen 40002, Thailand; nuapat@kku.ac.th; 3 Department of Pathology, Faculty of Medicine, Khon Kaen University, Khon Kaen 40002, Thailand; 4 Department of Pathology, Faculty of Medicine, Ramathibodi Hospital, Mahidol University, Bangkok 10400, Thailand; suchin.wor@mahidol.ac.th

**Keywords:** HPV, EBV, OSCC, miR-145, DNMT3B, methylation

## Abstract

Down-regulation of tumor-suppressive miR-145 has been reported in various malignancies, including oral squamous-cell carcinoma (OSCC) that is influenced by several factors, including Epstein-Barr virus (EBV) and human papillomavirus (HPV). Oncoviruses can modulate the expression of cellular microRNAs. Therefore, we sought to investigate the association of miR-145 down-regulation in OSCC with EBV and/or HPV infection, which might be a possible mechanism of these viruses in oral carcinogenesis. Herein, prevalence of EBV, HPV, and their co-infection was significantly higher in tumors than normal tissues of OSCC. EBV infection alone or jointly with HPV was significantly associated with down-regulation of miR-145 in tumors compared with normal adjacent tissues. In cell lines infected with EBV or HPV, miR-145 was also down-regulated. Consistently, methylation of miR-145 was significantly greater in tumors, and well correlated with increased expression of DNMT3B, which was influenced by infection with EBV and HPV. In cell lines, only EBV infection was associated with increased expression of DNMT3B. Moreover, the level of EBV-LMP1 mRNA in tumors was negatively correlated with miR-145 and positively correlated with DNMT3B. Therefore, EBV alone or jointly with HPV is associated with down-regulation of miR-145 and may influence on miR-145 promoter methylation through the induction of DNMT3B in OSCC.

## 1. Introduction

Oral squamous-cell carcinoma (OSCC) is the most common subtype of head and neck squamous-cell carcinoma (HNSCC) and the most frequently occurring malignancy in the oral cavity, representing more than 90% of all cancer types at this anatomical site. Over 300,000 new OSCC cases are diagnosed annually [1,2]. Development of OSCC is influenced by various risk factors, such as tobacco smoking, alcohol consumption, and betel quid chewing [3]. Infection by oncogenic viruses, in particular human papillomavirus (HPV) and Epstein-Barr virus (EBV), is also considered a risk factor for OSCC [4,5]. The incidence of EBV- and HPV-associated OSCC has been increasing recently. Joint infection with EBV and HPV is an important etiologic factor of this cancer [6]. HPV-positive OSCC tissues are often co-infected with EBV [7]. In contrast to HPV, it remains unclear whether EBV has a role in OSCC pathogenesis, and little is known about the pathogenesis of OSCC in cases of joint infection with EBV and HPV.

Accumulating evidence suggests that EBV and HPV can epigenetically regulate host gene expression to facilitate their life-long persistent infection and pathogenesis [8,9]. The viruses can disrupt cellular DNA-methylation machinery via their oncoproteins and can also regulate host microRNA expression [10,11]. MicroRNAs (miRNAs), non-coding, small, single-stranded RNAs, act as post-transcriptional regulators of gene expression, and are important in cellular processes [12]. Recently, down-regulation of miR-145 has been reported in various cancers. This miRNA was identified as a core miRNA of HPV-associated cervical cancer and HNSCC [13]. The miR-145 is tumor-suppressive and inhibits cell migration, invasion, and proliferation and induces apoptosis through targeting 3′UTR of its downstream targets [14,15]. A meta-analysis revealed that down-regulation of miR-145 was associated with an unfavorable overall survival in various malignancies and suggested that down-regulation of miR-145 could be used as a potential prognostic biomarker [16,17]. However, there is little information concerning whether down-regulation of miR-145 is associated with EBV and HPV infection in OSCC. Therefore, in the present study, we aimed to investigate the association of miR-145 expression in OSCC with EBV infection alone or co-infection with HPV, and a possible mechanism by which EBV and HPV infection influence on the expression of miR-145.

## 2. Materials and Methods

### 2.1. Study Design and Specimen

A total of 84 formalin-fixed paraffin-embedded (FFPE) OSCC samples, diagnosed at Srinagarind Hospital during 2012–2015, were retrieved from archived material in the Department of Pathology, Faculty of Medicine, Khon Kaen University, Thailand. FFPE tissues were cut, and tumor and normal adjacent tissues were separated using laser-capture microdissection. Buccal cells were collected from control subjects without cancer or any chronic infection in the head and neck. The buccal cells were collected using conical cytobrush by scratching at the site of buccal cavity of the control subjects. After sampling, the cytobrush was placed in a tube containing phosphate buffered saline (PBS) and centrifuged at 2000× *g* for 10 min [18,19]. The use of FFPE OSCC archived specimens and buccal cells was approved by the Khon Kaen University Ethics Committee for Human Research (No. HE581437 and No. HE561407, respectively). Informed consent was obtained in writing from all participants prior sample collection.

In the present study, the tumor and normal adjacent tissues of OSCC samples were used to determine the presence of EBV and HPV and to examine the expression levels of miR-145, DNMT1, DNMT3B and the methylation status of miR-145. The expression levels of miR-145, DNMT1 and DNMT3B were confirmed in cell lines either infected with or without EBV and HPV and normal human tongue keratinocyte cell lines. The microarray data of HNSCC was retrieved from the Gene Expression Omnibus (GEO) database to examine whether miR-145 is down-regulated in tumor tissues, especially in the presence of virus infection.

### 2.2. Cell Lines

Cell lines used: human OSCC cells (ORL-48(T) and ORL-136(T) (kindly provided by Prof. Dr. Sok Ching Cheong, Cancer Research Initiatives Foundation, Sime Darby Medical Centre Jaya, Malaysia); SCC25 and HPV16-positive SCC90 and human skin squamous-cell carcinoma (HSC1) cells (kindly provided by Dr. Tohru Kiyono, National Cancer Center (NCC), Chiba, Japan); EBV-positive cells (SCC25-EBV and HSC1-EBV cells) previously established [20], and cervical cancer-cell lines (HPV-negative C33A, and HPV-positive SiHa, CaSki, and HeLa cells). Cells were maintained in Dulbecco’s Modified Eagle Medium/F12 (DMEM/F12; Gibco, Breda, The Netherlands) and supplemented with 10% fetal bovine serum (Gibco) and antibiotics (Nacalai Tesque Inc., Kyoto, Japan). Human tongue keratinocyte cell lines, HTK1-EGFP and HTK1-16E6E7 (kindly provided by Dr. Tohru Kiyono) were cultured in Keratinocyte-SFM medium (Gibco) and supplemented with bovine pituitary extract, epidermal growth factor and antibiotics (Nacalai Tesque Inc., Kyoto, Japan). All cell lines were cultured at 37 °C in a humidified incubator with 5% CO_2_.

### 2.3. Detection of HPV and EBV Infection in OSCC Tissues

DNA was extracted from FFPE tissues using a DNeasy Blood and Tissue Kit (QIAGEN, Hilden, Germany) according to the manufacturer’s instructions. The extracted DNA was checked for quality by spectrophotometry and by amplification of the GAPDH gene using real-time polymerase chain reaction (RT-PCR).

Consensus primers for the conserved L1 ORF of HPV; GP5+/GP6+ were used for HPV detection and primers targeting BALF5 were used to screen the presence of EBV DNA by RT-PCR on a LightCycler^®^ 480 instrument (Roche, Penzberg, Germany [21,22]). To determine the HPV genotype, reverse line blot hybridization (RLBH) was performed [23]. To confirm EBV infection, quantitative reverse-transcription PCR (qRT-PCR) was used to examine the expression of EBV-encoded small RNA 1 (EBER1) and latent membrane protein 1 (LMP1). Total RNA was extracted from tumor and normal adjacent tissues using RNeasy FFPE kit (QIAGEN) according to the manufacturer’s instructions. Total RNA, 1 µg, was used for cDNA synthesis using SuperScript^®^ III First-Strand Synthesis (Invitrogen, Carlsbad, CA, USA) according to the manufacturer’s instruction. A tissue was regarded as positive for EBV if amplification was successful for both EBER1 and BALF5. The primers used in this study are listed in Table 1.

### 2.4. Detection of miR-145 and DNMTs Expression by qRT-PCR

The expression of miR-145, DNMT1, and DNMT3B was determined by qRT-PCR [24,25] and was performed in triplicate on an Applied Biosystems QuantStudio6 instrument (Thermo Scientific, Waltham, MA, USA). The expression of miR-145 was evaluated by the comparative CT method and normalized using U6 snRNA as the endogenous control. The expression of DNMT1 and DNMT3B was also evaluated by qRT-PCR and normalized using GAPDH as the endogenous control. The sequences of primers used in this study are listed in Table 1. Relative expression levels of miR-145, DNMT1, and DNMT3B were examined by the 2^−ΔΔCT^ method.

### 2.5. Methylation Status of miR-145 Promoter Determined by Methylation-Specific Real-Time PCR (MSP)

The extracted DNA from tumor and normal adjacent tissues was subjected to bisulfite conversion using Epitect^®^ Fast DNA Bisulfite kit (QIAGEN) and the procedure was performed according to the manufacturer’s instructions. The bisulfite-converted genomic DNA was amplified using primers for the unmethylated and methylated reactions ([26], Table 1). The MSP was performed by real-time PCR assay using SsoAdvanced^TM^ SYBR^®^ Green Supermix (Bio-Rad, Hercules, CA, USA) in the QuantStudio 6 Flex Real-Time PCR System (Applied Biosystems, Foster City, CA, USA). The primers for MSP were designed to distinguish methylated from unmethylated DNA with different lengths of the amplicons, 98 and 84 bp, respectively. PCR products were electrophoresed through a 2% agarose gel and visualized by using a gel documentation system (Bio-Rad).

### 2.6. Statistical Analysis

Data are expressed as mean ± SD (standard deviation). The symbols *, ** and *** denote statistically significant differences as *p* < 0.05, 0.01 and 0.001, respectively. Statistical analysis was performed using the Graphpad Prism software. The prevalence of EBV and HPV infection and the correlation of virus infection in tumor and normal adjacent tissues were analyzed using chi-square tests. A Mann-Whitney U test was used to test whether there was a difference between two independent groups.

## 3. Results

### 3.1. Patient Characteristics

The archived specimens were obtained from 84 OSCC patients who had the characteristics as shown in Table 2; their mean age was 61 years and 48.8% were male. The tongue was the most common anatomical site of OSCC and the floor of mouth was the least common. Well-differentiated tumors were the most common histological grade (54.5%), followed by moderately and poorly differentiated tumors, respectively.

### 3.2. Prevalence of HPV, EBV, and Their Co-Occurrence in OSCC

**Figure 1:**
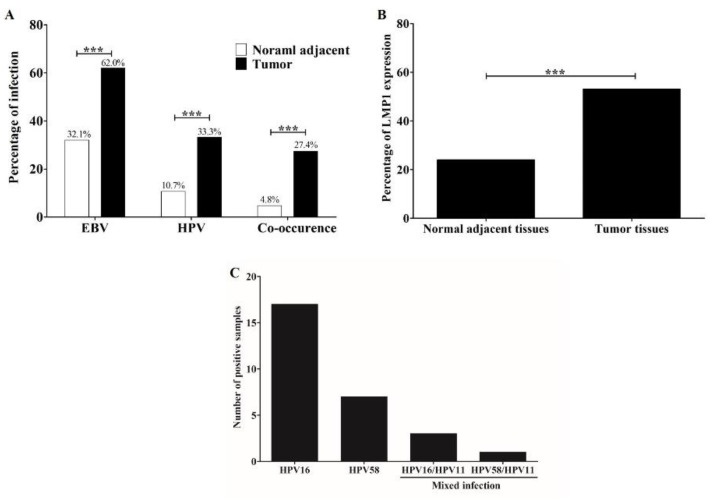
EBV and HPV infection is more frequently detected in OSCC tumor tissues than in normal tissues. Prevalence of HPV, EBV, and their co-occurrence in tumor and normal adjacent tissues (**A**). The LMP1 expression in tumor and normal adjacent tissues was determined by qRT-PCR (**B**). Distribution of HPV genotypes in OSCC tissues as determined by RLBH (**C**) ***: *p* < 0.001.

Prevalence of EBV, HPV, and their co-occurrence was examined in 84 pairs of tumors and normal adjacent tissues by qRT-PCR. EBV was significantly detected in tumor (62.0%) than in normal adjacent (32.1%) tissues (Figure 1A). Prevalence of HPV in tumor tissues (33.3%) was significantly higher than normal adjacent tissues (10.7%, Figure 1A). In addition, these viruses co-occurred in 27.4% of tumor tissues and only in 4.8% of normal adjacent tissues (Figure 1A). Among EBV-positive cases, LMP1 expression was detected in 53.1% and 24.0% of tumor and normal adjacent tissues, respectively (Figure 1B). HPV16 was most commonly found in HPV-positive OSCC samples (60.7%, Figure 1C). These results showed the association of EBV, HPV, and their co-concurrence with OSCC and suggest that these viruses play a role in oral carcinogenesis.

### 3.3. EBV and HPV Modulate miR-145 Expression in OSCC

**Figure 2:**
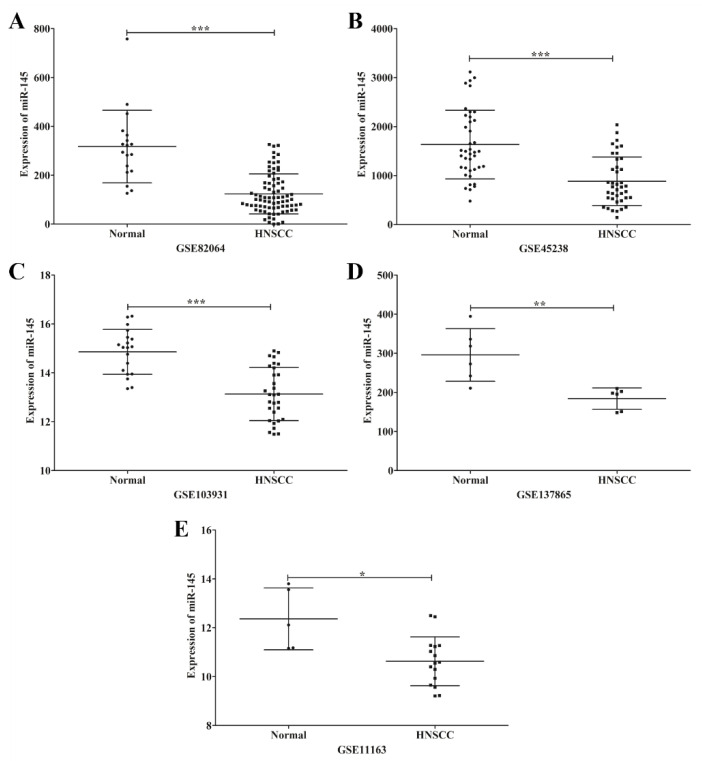
Representative scatter plots of miR-145 expression in normal and HNSCC tissues in microarrays. Expression levels of miR-145 in normal and HNSCC tissues plotted from microarray data; GSE82064 (**A**), GSE45238 (**B**), GSE103931 (**C**), GSE137865 (**D**) and GSE11163 (**E**). *: *p* < 0.05; **: *p* < 0.01; ***: *p* < 0.001.

**Figure 3:**
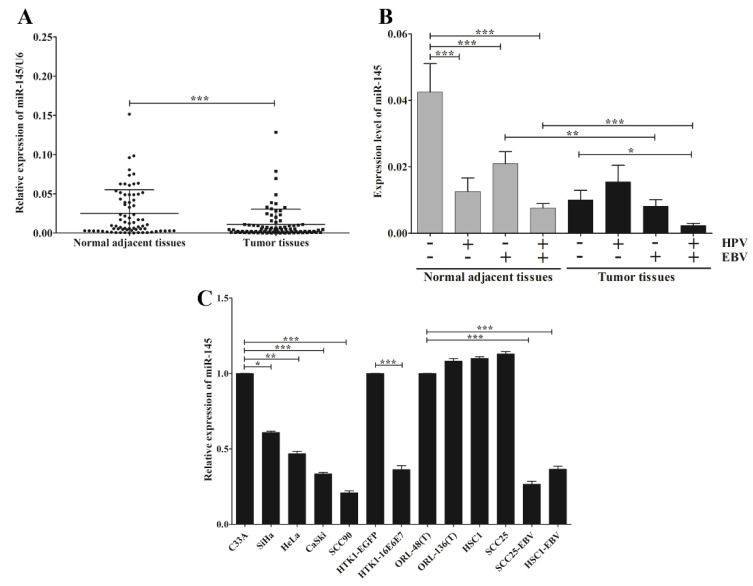
EBV infection alone or jointly with HPV associates with the down-regulation of miR-145 in OSCC. The expression of miR-145 in OSCC tissues (**A**) and OSCC tissues with or without EBV and/or HPV (**B**) and in cancer cell lines (**C**) was examined by qRT-PCR. *: *p* < 0.05; **: *p* < 0.01; ***: *p* < 0.001.

MicroRNA-145 was down-regulated in various cancer cell lines, including HNSCC. To further confirm this result, microarrays from the GEO Dataset using HNSCC and non-cancerous tissues were obtained and analyzed for miR-145 expression. This result demonstrated that miR-145 was significantly down-regulated in HNSCC tissues compared with non-cancerous tissues (Figure 2). Furthermore, we examined miR-145 expression in OSCC tissues using qRT-PCR. As expected, miR-145 was significantly down-regulated in tumor tissues compared with normal adjacent tissues (Figure 3A).

Accumulating evidence suggests that HPV and EBV modulate expression patterns of host genes, including miRNAs. Therefore, we analyzed the correlation between miR-145 and EBV and/or HPV infection. Figure 3B shows the effects of EBV and HPV infection, individually or jointly, on the down-regulation of miR-145 in tumor tissues and normal adjacent tissues. Interestingly, the greatest reduction of miR-145 expression in tumor tissues was significantly associated with joint infections of EBV and HPV, but not with HPV infection alone (Figure 3B). This result suggests that infection of EBV jointly with HPV has the synergistic effect to modulate miR-145 expression in OSCC. We also examined whether EBV and HPV are associated with down-regulation of miR-145 in a range of cancer cell lines. Here, miR-145 was significantly down-regulated in HPV-positive cancer cell lines (SiHa, HeLa, CaSki and SCC90) relative to HPV-negative cancer cells (Figure 3C). MicroRNA-145 was also significantly down-regulated in normal human tongue keratinocytes expressing HPV16E6E7 (HTK1-16E6E7) relative to than HTK1-EGFP cells (Figure 3C), suggesting that HPV infection modulates miR-145 expression via viral oncogenes. In addition, miR-145 was down-regulated in EBV-positive cells when compared with EBV-negative cells (Figure 3C). These results demonstrate that EBV and HPV infection dysregulate the expression of miR-145 in OSCC.

### 3.4. EBV and HPV, Separately or Jointly, Associate with the Silencing of miR-145 via DNA Hypermethylation

**Figure 4:**
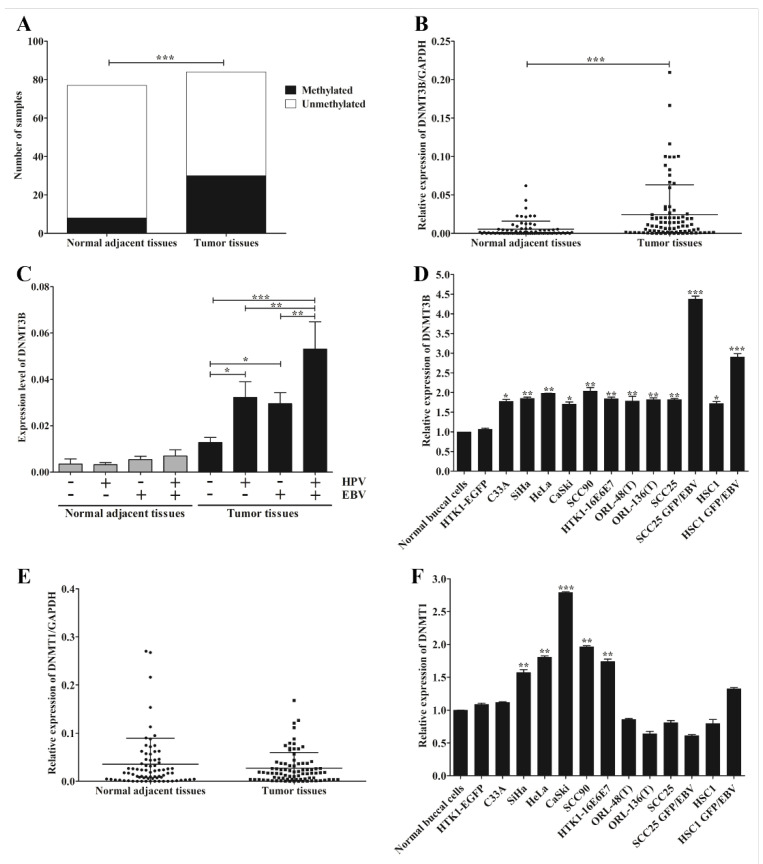
EBV silences miR-145 by DNA methylation via DNMT3B. The methylation status of miR-145 was determined by MSP using specific primers. The number of samples in which hypermethylation occurred in tumor and normal adjacent tissues (**A**). The expression of DNMT3B in OSCC tissues (**B**), OSCC tissues with or without the infection of EBV and/or HPV (**C**) and cancer cell lines (**D**) were examined by qRT-PCR. The expression levels of DNMT1 in OSCC tissues (**E**) and cell lines (**F**) were examined by qRT-PCR. *: *p* < 0.05; **: *p* < 0.01; ***: *p* < 0.001.

We observed a correlation between infection of EBV or HPV and down-regulation of miR-145 in OSCC. There is still limited information on the possible mechanism by which they achieve this. However, DNA methylation is a likely candidate mechanism. To determine whether down-regulation of miR-145 is associated with DNA hypermethylation, MSP was performed. The result showed that DNA methylation of miR-145 was significantly greater in tumor tissues (35.7%) than in normal adjacent tissues (8.0%, Figure 4A and Figure S1). This result suggests that down-regulation of miR-145 in OSCC may, at least in part, be regulated by DNA methylation.

Previously, DNMT3B was identified as a target of miR-145 and also regulated miR-145 expression via a negative feedback loop mechanism [27]. To further support our result that down-regulation of miR-145 is mediated via DNA methylation, we examined the expression of DNMT3B in OSCC tissues and various cancer cell lines by qRT-PCR. DNMT3B expression was significantly up-regulated in tumor tissues (Figure 4B) and this up-regulation was significantly associated with viral infection (Figure 4C). DNMT3B was significantly up-regulated in all cancer cell lines, especially in EBV-positive cell lines (Figure 4D). In addition, we also examined the expression of DNMT1, a maintenance DNA methyltransferase enzyme, in OSCC tissues and cell lines by qRT-PCR. In contrast, DNMT1 expression in tumor tissues was not significantly different from that in normal adjacent tissues (Figure 4E). However, it was significantly up-regulated in HPV-positive cell lines, but not EBV-positive cells (Figure 4F). Collectively, these results suggest that infection with EBV or HPV modulates the expression of DNMT3B, which may function in the methylation of miR-145 in OSCC.

**Figure 5:**
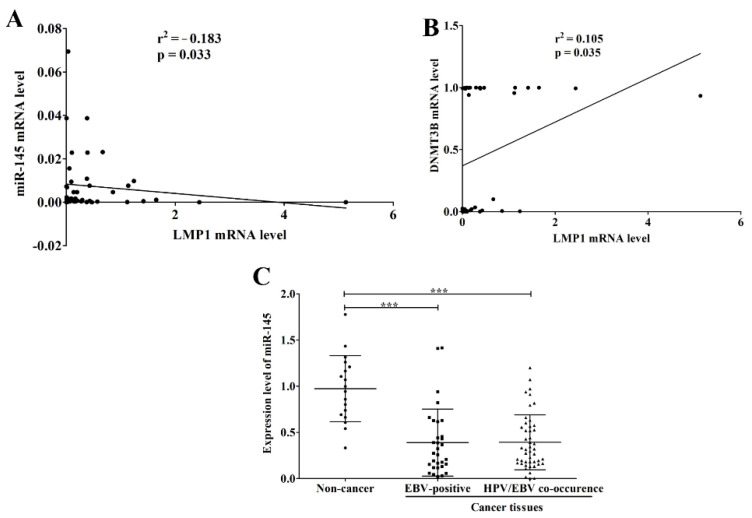
EBV infection is associated with the down-regulation of miR-145. The correlation of LMP1 mRNA level with miR-145 (**A**) and DNMT3B (**B**) mRNA levels was analyzed by Pearson correlation. Expression data of miR-145 in normal and HNSCC tissues, that were obtained from microarray data (GSE82064), were analyzed and plotted according to the infection of EBV and/or HPV (**C**). ***: *p* < 0.0001.

As shown here, EBV infection was significantly associated with the up-regulation of DNMT3B. A previous study demonstrated that LMP1 up-regulates the expression of DNMT3B in NPC cells through NF-κB pathway [28]. We therefore analyzed the correlation of LMP1 mRNA level with miR-145 and DNMT3B mRNA levels. As expected, the LMP1 mRNA level was negatively correlated with miR-145 expression and positively correlated with DNMT3B expression (Figure 5A,B). In addition, microarray data of HNSCC were obtained from the GEO Dataset and analyzed. As expected, miR-145 was significantly down-regulated in HNSCC tissues infected with EBV alone or jointly with HPV and EBV when compared to normal tissues (Figure 5C). Collectively, these results suggest that infection with EBV, alone or jointly with HPV, down-regulates miR-145 expression in OSCC tissue via de novo hypermethylation by induction of DNMT3B.

## 4. Discussion

EBV and HPV are tightly associated with various malignancies via epigenetic regulators, such as miRNAs, which are also frequently modulated in cancers, especially in virus-associated cancers, and are strongly associated with DNA methylation [29]. MicroRNA-145 is a tumor-suppressive miRNA that is down-regulated in cancers, such as cervical cancer [30] and OSCC [31]. The relationship between oncogenic viral infections and down-regulation of miR-145 in OSCC has not been studied previously; rectifying this situation was the aim of our study. Our finding was consistent with previous studies showing that miR-145 was generally down-regulated in cancer cell lines and tumor tissues. Accumulating evidence has demonstrated that down-regulation of miR-145 significantly promotes cancer progression: conversely, up-regulation of miR-145 significantly inhibits cancer progression by targeting cellular pathways, such as Wnt/β-catenin, PI3K/AKT and EGFR [32,33]. In OSCC, overexpression of miR-145 dramatically inhibits cancer progression via c-Myc and CDK6. Down-regulation of miR-145 was suggested to be a diagnostic and therapeutic target in cases of OSCC [34].

As mentioned earlier, miR-145 has been identified as a core miRNA associated with HPV infection. This miRNA is down-regulated by high-risk HPV31E7. To advance our knowledge, we further examined the relationship between EBV and HPV infection on the one hand with the expression and methylation of miR-145 on the other. Consistently, we also found that miR-145 was down-regulated in cells expressing HPV16E6E7 and in HPV16-positive OSCC cells. Similarly, miR-145 was dramatically down-regulated in virus-infected cells (Figure 2B). This is consistent with a previous study which demonstrated that EBV down-regulates miR-145 expression in EBV-associated cancers via the function of EBNA3A and EBNA3C by silencing the miR-143/miR-145 cluster [35]. We found that miR-145 was significantly down-regulated in OSCC tumor tissues infected with both EBV and HPV. Thus, EBV and HPV joint infection might be one factor that plays a role in the down-regulation of miR-145 in OSCC.

Previous studies demonstrated that the expression of miR-145 was also down-regulated in OSCC tissues [31,34,36]. Consistently, in the present study, the expression of miR-145 was decreased in OSCC tumor tissues when compared with normal adjacent tissues, especially in the presence of EBV and HPV. The infection of EBV and/or HPV might indirectly regulate the expression of miR-145. DNA methylation is one of the epigenetic mechanisms that modulates the expression of genes including non-protein coding genes. Therefore, we hypothesized that down-regulation of miR-145 might be brought about by DNA methylation. Previous studies have demonstrated that miR-145 is epigenetically silenced by DNA methylation in various cancers [37,38]. According to our finding, miR-145 was hypermethylated in OSCC tissues, although only in 35.71% of these tissues. Therefore, other mechanisms and epigenetic mechanisms that are induced by viruses or risk factors may play a role in the regulation of miR-145. Previously, down-regulation of miR-145 was also shown to be mediated by histone methylation in prostate cancer cell lines [39] and colorectal cancer cell lines [40]. In addition, the expression of miR-145 could be regulated by other non-coding RNAs, called long non-coding RNAs (lncRNAs). Previous studies have shown that some lncRNAs, such as TUG1, UCA1, and AFAP1-AS1, regulated the expression of miR-145 by a sponging mechanism in glioma stem cells (GSCs) [41], nasopharyngeal carcinoma (NPC) cell lines [42], and OSCC cell lines [43], respectively. In addition, miR-145 can be regulated by circular RNA (circRNA), non-coding RNA. Recently, Su and colleagues demonstrated that circDNM3OS regulated miR-145 in cholangiocarcinoma cell lines by sponging to miR-145 [44]. Collectively, the down-regulation of miR-145 may, at least in part, be mediated through DNA methylation.

We found that hypermethylation and down-regulation of miR-145 was significantly associated with EBV and HPV infection in OSCC. Furthermore, the expression levels of miR-145 and DNMT3B were negatively and positively correlated with LMP1 mRNA level, respectively. A previous study demonstrated that the LMP1 can up-regulate the expression of DNMT3B in NPC cells via NF-κB signaling pathway [26]. In addition, previous studies have demonstrated that EBV infection of B lymphocytes can induce de novo DNA methyltransferases, but not DNMT1 [45]. In epithelial cancers, LMP1 indeed mediates the expression of host genes via DNA methylation through up-regulation of DNMT1, DNMT3A and DNMT3B, which further promotes the migration of cancer cells [46,47]. These results suggest that EBV jointly with HPV may regulate miR-145 in OSCC through DNA methylation via the induction of DNMT3B.

## 5. Conclusions

In conclusion, our findings indicated the association of HPV and EBV infection with down-regulation of miR-145 and suggest that infection of EBV alone or jointly with HPV may play an important role in the development of OSCC, at least in part by regulation of miR-145 expression through DNA methylation via the activation of DNMT3B. However, further study is required regarding the underlying mechanism by which HPV and EBV down-regulate miR-145 via DNA methylation.

## Figures and Tables

**Table 1:** Primer sequences.

Gene Name	Forward (5′-3′)	Reward (5′-3′)
Methylated-miR-145	GGGTTTTCGGTATTTTTTAGGGTAATTGAAGTTTC	TAAAATACCACACGTCGCCG
Unmethylated-miR-145	GGGTTTTTGGTATTTTTTAGGGTAATTGAAGTTTT	AACCAAAATAAAATACCACACATCACCA
GP5+/GP6+	TTTGTTACTGTGGTAGATACTAC	GAAAAATAAACTGTAAATCATATTC
*BALF5*	CGAGTCATCTACGGGGACACGGA	AGCACCCCCACATATCTCTTCTT
*EBER*	CTACGCTGCCCTAGAGGTTTT	CAGCTGGTACTTGACCGAAGA
*LMP1*	TCCTCCTCTTGGCGCTACTG	TCATCACTGTGTCGTTGTCC
*miR-145*	ATCGTCCAGTTTTCCCAGG	CGCCTCCACACACTCACC
*DNMT1*	TACCTGGACGACCCTGACCTC	CGTTGGCATCAAAGATGGACA
*DNMT3B*	GGCAAGTTCTCCGAGGTCTCTG	TGGTACATGGCTTTTCGATAGGA
*GAPDH*	TCATCAGCAATGCCTCCTGCA	TGGGTAGCAGTGATGGCA

**Table 2:** Demographic characteristics of OSCC patients.

Demographic Features	OSCC Tissues
	(*n* = 84)
Age, year	60.65
Range, year	27–90
Gender	
Male	41
Female	31
ND	12
Site of OSCC	
Tongue	42
Lip	4
Buccal mucosa	8
Gum	5
Floor of mouth	1
Palate	10
Gingiva	2
ND	12
Histological grades	
Well differentiated	46
Moderately differentiated	15
Poorly differentiated	3
ND	20

ND: Not determined.

## Data Availability

The published article includes all datasets generated or analyzed during this study.

## Supplementary Materials

The following are available online at https://www.mdpi.com/article/10.3390/microorganisms9122496/s1, Figure S1: An agarose gel showing amplified methylated and unmethylated fragments from representative samples of tumor and normal adjacent tissues.
