# Supplementary material for: Epstein-Barr Virus Infection Alone or Jointly with Human Papillomavirus Associates with Down-Regulation of miR-145 in Oral Squamous-Cell Carcinoma
Source: Microorganisms. 2021 Dec 2;9(12):2496. doi: 10.3390/microorganisms9122496 (PMC8708579; doi:10.3390/microorganisms9122496)
Supplement: Supplementary file 1 [file microorganisms-09-02496-s001.zip › microorganisms-1471777-supplementary.pdf]

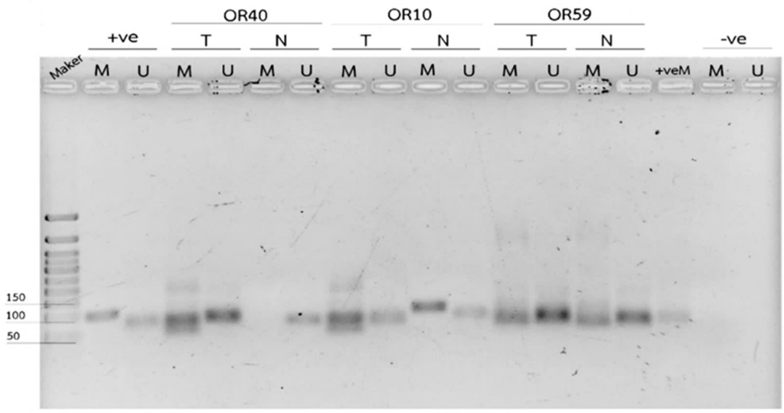

Figure S1. An agarose gel showing amplified methylated and unmethylated fragments from representative samples of tumor and normal adjacent tissues. The length of the amplicon of methylation and unmethylation was 98 and 84 bp, respectively. T: tumor tissue, N: normal adjacent tissue, M: methylated and U: unmethylated.
